# Hope-Based Program for Portuguese Outpatients with Advanced Chronic Illness in a Community Setting: A Randomized Control Trial

**DOI:** 10.3390/ijerph20021566

**Published:** 2023-01-14

**Authors:** Ana Querido, Carlos Laranjeira

**Affiliations:** 1 School of Health Sciences, Polytechnic of Leiria, Campus 2, Morro do Lena, Alto do Vieiro, Apartado 4137, 2411-901 Leiria, Portugal; 2 Centre for Innovative Care and Health Technology (ciTechCare), Polytechnic of Leiria, Campus 5, Rua de Santo André-66-68, 2410-541 Leiria, Portugal; 3 Center for Health Technology and Services Research (CINTESIS), NursID, University of Porto, 4200-450 Porto, Portugal; 4 Comprehensive Health Research Centre (CHRC), University of Évora, 7000-801 Évora, Portugal

**Keywords:** randomized controlled trial, hope, comfort, quality of life, palliative care

## Abstract

Background: Hope is widely considered a subjective phenomenon able to bring beneficial consequences to human health and existence. Maintaining hope amid a life-threatening disease and during palliative care is critical. The study aims to examine the effectiveness of a psychosocial supportive Hope Promotion Program (HPP) in enhancing hope, comfort, and quality of life in Portuguese adult outpatients with advanced and progressive chronic illness. Method: Using a parallel Randomized Control Trial (RCT) with pre-post design, 56 cancer outpatients from two day hospitals. Participants were randomly assigned to either a control group (n = 28) or an intervention group (n = 28). The primary outcome measure was hope. Secondary measures included comfort and quality of life. Participants were assessed at baseline, day 15, and day 30 of follow-up. Results: Baseline characteristics were similar between the two groups. In the intervention group, there was a significant increase in the total hope scores after the HPP (day 15). Significant differences were still present after one month (*p* < 0.05). There was also a significant increase in comfort and quality of life scores in the intervention group one month after HPP (*p* = 0.018). Conclusions: The HPP may be an effective intervention to increase hope and improve comfort and quality of life among palliative patients. Future studies should increase sample size, diversify settings, and include longer and more detailed follow-ups.

## 1. Introduction

Palliative care, as a medical speciality, has grown to occupy an essential area between the opposing emphases on prolonging life and hastening death [1]. For those undergoing palliative care, hope is a critical psychosocial resource [2,3,4], essential for having a meaningful life and a peaceful death [5]. People receiving palliative care are more hopeful and experience the possibility of a better future [6]. According to studies in palliative and end-of-life care, patients use *hope* as a noun to express a personal, individual construct but use *hope* as a verb (such as *hoping to live*) to express an interpersonal construct [7]. This relational co-construction of hope “can increase the potential for uncertainty and abstraction even further, all the while maintaining the positive meaning-making allure of being hopeful” [6] (p. 1). Recently, a synthesis of review studies stressed that “hope was conceptualized as an expectation (appraisal of a future outcome), resilience (endurance of adversity), and a desire (expression of meaning)” [8] (p. 197).

The promotion and inspiration of hope is closely linked to the effectiveness of nursing practice [9,10], and is thus an essential component in clinical practice. Nurses, due to their proximity to patients and families, are in a strategic position to influence hope, both through its promotion and reduction [11,12].

Several studies have found that a range of psychosocial interventions can help palliative care patients feel more hopeful [13,14,15,16,17]. These interventions may include: (a) forgiveness therapy [18], (b) dignity therapy [19], (c) short-term life review activities [20], (d) opening a new palliative day care program [21], and (e) the Living with Hope Program [2,22]. Although research has shown that hope-based interventions are helpful, the link between these interventions and their outcomes is still poorly understood [17,23]. Moreover, since hope is a multidimensional, dynamic, culturally sensitive and individualized process [24], it is not possible to guarantee that these interventions will have the same impact in the Portuguese context without a prior cultural adaptation process.

The evaluation and promotion of hope is one of the standards of good clinical practice in palliative care [25]. Similarly, the most recent perspective on evaluating quality of nursing care includes comfort, hope and resilience as positive outcomes [17,26]. Since the objective of providing holistic care in end-of-life situations is to promote comfort and Quality of Life (QoL), it is important to operationalize and measure the impact of interventions on these variables. However, to date, there were no validated interventions in Portugal to promote hope, comfort or QoL that were specifically developed for individuals in palliative situations.

The overall aim of the present study was to evaluate the effectiveness of a psychosocial program to promote hope, comfort and QoL in Portuguese adult outpatients with advanced and progressive chronic illness. Understanding the effects of such programs will provide insights for other interventions focused on fostering hope in palliative care patients.

## 2. Materials and Methods

### 2.1. Study Design

This was a two-group, parallel Randomized Control Trial (RCT), with a pre-test post-test control group design and repeated post-test measures. This RCT was retrospectively registered in the United States of America Clinical Trials Registry Platform (NCT02723799) according to the investigation protocol (see Table 1).

### 2.2. Setting and Participants

Participants were recruited from the day hospitals of two medical institutions in the central region of Portugal. The services in both contexts are especially dedicated to providing healthcare to chronic patients at different stages of the disease, including the palliative phase, in a clinic with less than 24 h access and surveillance. After the main researcher (AQ) explained the study, each participant signed a written consent form before participating in the study. Consolidated Standards of Reporting Trials (CONSORT) guidelines were followed throughout the study [27].

#### 2.2.1. Eligibility Criteria

After agreeing to participate, participants were thoroughly screened for eligibility by the main researcher (palliative care and mental health nurse). Screening involved completion of the Mini-Mental State Examination (MMSE) [28] and the Karnofsky Performance Scale (KPS) [29], as well as asking about the presence of uncontrolled symptoms. Key inclusion criteria were: (1) adult over 18, diagnosed with advanced and progressive chronic disease; (2) clinical indicators of advanced disease: disease instability, decreased response to treatments and metastatic cancer; and (3) ability to speak and comprehend Portuguese. Exclusion criteria included: (1) cognitive impairment (assessed by the Portuguese version of the MMSE [28]) precluding providing informed consent; (2) the presence of uncontrolled symptoms (nausea, vomiting, pain); and (3) functional status below 30 (assessed by the KPS), as these patients are particularly vulnerable and unable to complete the study. Following this screening, non-eligible participants were immediately told they were not eligible for the study and were thanked for their time.

#### 2.2.2. Random Assignment

The principal investigator (AQ) randomly allocated eligible participants to one of the two group using blocks of eight individuals, in a 1:1 distribution system, in order to guarantee an approximate number of individuals allocated to each group. Thus, for each block of eight individuals, a white ball/black ball system was used, using four balls of each colour for this purpose.

### 2.3. Intervention—Hope Promotion Program (HPP)

The intervention was designed based on the Narrative Communication Model of Hope Seeking Intervention [30]. This model is “based on the ideas that (a) hope as an experience is only possible through a person’s existential quest and (b) nurses or professionals help clients in their journey to find hope” [30] (p. 3). Faced with advanced and progressive chronic disease, people live one day at a time, based on the individual’s perception of hope, in a timeline directed towards the future and the achievement of goals, with death as the ultimate limit. This model comprises the attributes of hope identified in the literature review: experiential, relational, rational and spiritual thinking processes [31]. In the context of terminal illness, this involves positive expectations, personal qualities, spirituality, goals, comfort, help/care, interpersonal relationships, control, legacy and life review [24].

The participants randomly assigned to the intervention group were given the individual-based intervention (HPP) in three home visitation sessions conducted by a nurse. Each session lasted between 90 min and 2 h 30 min, depending on the patient’s need, capacity and will. The interval between sessions was scheduled to last two to three days, so that the full intervention occurred over 10 days. The possibility of adjusting session times according to the client’s conditions was considered whenever complications associated with the disease or treatments prevented the session. Participants were able to have a family member present whenever they wished, which happened in half of the situations. Each participant had an equal number of sessions with the same predetermined objectives.

The first session, with the objective of awakening the self-perception of hope and expressing it verbally, was accompanied by viewing a Portuguese adaptation of the “Living with hope” video [32]. This video depicts a discussion between terminally ill people and their families about their experience in keeping hope alive. Such visualizations allow participants to empathize with those depicted in the video and change their views with regard to their own ability to perform certain tasks (video modelling) [32,33].

The second session was focused on the expression of feelings and emotions related to the experience of the disease, and on working positively on the client’s capacities to carry out hope-promoting activities. An activity guide was provided to the patient and was explored during the session. Some hope-focused activities were proposed, such as gratitude exercises, therapeutic and forgiveness letters (writing someone a letter), a hope album (remembering memories of hope from the past), or stories of the present and a hope kit (collecting objects that are hopeful and significant) [34]. Participants were assisted in choosing the hope activity with which they felt comfortable, and which was achievable and appropriate to their situation and condition.

The third session’s main objective was to facilitate the transcending of the suffering associated with the advanced disease through teaching and training relaxation using mental images [35]. In this session, the nurse helped the participant express opinions and feelings related to the activity and reinforced the ability to deal with the situation of advanced disease through the realization of a plan to incorporate the chosen activity into their daily routine.

### 2.4. Sample Size

The sample size was calculated using power analysis based on four components: the level of significance or alpha (α), sample size, population effect size (ES), and power (1-β) [36]. Power tables indicate the necessary sample size for a t-test to detect a significant difference between two independent samples of equal size, drawn from normal populations and assuming equal variances. The estimated sample size for each group was 26, using a conservative alpha level (0.6) and allowing the detection of a 0.1 effect size with a power of 0.70. This sample size was confirmed using the minitab power calculator [www.minitab.com] (accessed on 10 September 2021).

Given the inherent characteristics of individuals with advanced chronic disease, and the expected missing data and high attrition of the study, all subjects who met the initial selection and agreed to participate in the study were included in the randomization process.

### 2.5. Outcome Variables and Measurements

The primary outcome was hope, as measured using the Portuguese version of the Herth Hope Index (HHI) validated for chronic conditions [37]. The HHI is a 10-item Likert scale, arranged with scores from 1 to 4, that describes two sub-scales of hope: (a) temporality, trust and interconnection; and (b) positive readiness and expectancy. The HHI takes approximately 5 min to complete. Global scores range from 10 to 40, with a higher score indicating more hope. The Cronbach’s alpha of the HHI was 0.87 [37].

Secondary outcomes were:

(a) Comfort, as measured by the Portuguese version of the Hospice Comfort Questionnaire (HCQ) [38], which is based on Kolcaba’s holistic comfort questionnaire [39]. The HCQ contains 26 items scored from 1 (strongly disagree) to 6 (strongly agree) points that measure holistic comfort defined as the immediate state of gaining strength by satisfying the need for relief, ease, and transcendence in four contexts of experience: physical, psychospiritual, sociocultural, and environmental [39]. Higher scores indicate higher reported comfort. The Cronbach’s alpha for this tool was previously reported as 0.86 [38].

(b) QoL, as measured by the Portuguese version of McGill Quality of Life Questionnaire (MQoL) [40] originally developed by Cohen et al. [41]. The MQoL evaluates four domains of QoL: physical health, psychological symptoms, existential well-being, and support. The MQoL is comprised of 16 items, assessed in a self-rating numerical scale (0–10) and a single item scale (0–10) to assess subjective QoL. Higher scores indicate better QoL. The reliability for this questionnaire was favourable, with a Cronbach’s alpha = 0.89 [40].

### 2.6. Data Collection Procedure

**Figure 1:**
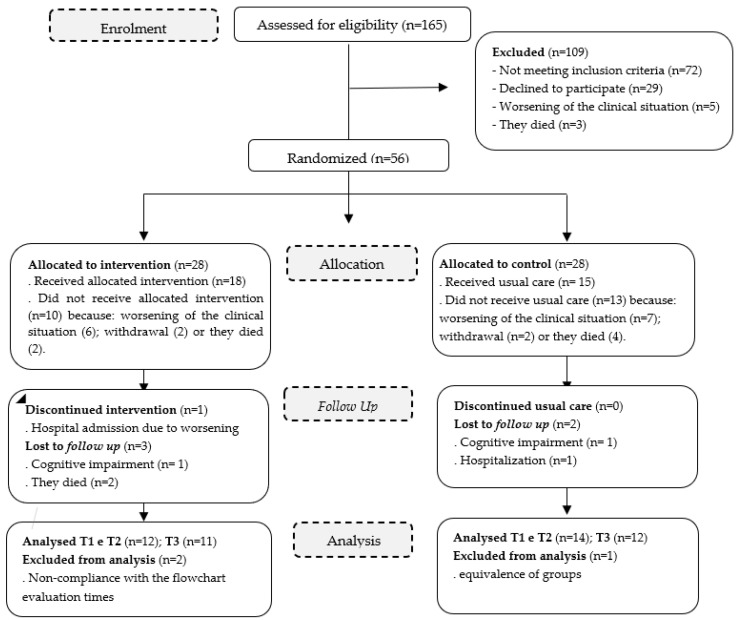
Participant flow through the phases of the RCT.

Data collection took place over an 8-month period. Participants completed baseline (T1) instruments upon enrolment. Follow-up instruments were collected immediately after the intervention (T2—15 days later), and one month after the program was completed (T3). Figure 1 outlines the study procedure.

Baseline data collected for all participants included: (1) demographics (gender, age, marital status and social status); (2) clinical characteristics (medical diagnosis, functional status, presence of medical symptoms, like pain, fatigue or depression); (3) the Herth Hope Index [HHI]; (4) the McGill Quality of Life Questionnaire [MQoL]; and (5) the Hospice Comfort Questionnaire [HCQ].

The intervention group received the HPP, and the control group received a standard care and palliative care approach provided locally by a multidisciplinary healthcare team. After the intervention (T2 and T3), participants from the two groups completed the HHI, MQoL and HCQ through a face-to-face interview by a research assistant. As participants were suffering from a severe illness, we made every effort to simplify survey completion, including reading the survey over the phone or during home visits. Most follow-up contacts were made by home visitation and telephone, with the remainder occurring during normal clinic appointments. All of the participants, care providers, and the research assistant who assessed the participant’s outcomes were blind to the intervention.

### 2.7. Analysis

A per-protocol analysis was done for all outcome variables and included only those patients who accomplished all assessments. Descriptive statistics were calculated to summarize patients’ characteristics and other baseline variables. The normality of distribution was checked using the Shapiro-Wilk test. Therefore, comparisons between the two study groups were analysed using the nonparametric Mann–Whitney test. The relationship between hope, comfort and QoL was tested using Spearman’s rho. Additionally, Wilcoxon tests were completed to compare pre-test and post-test scores between groups. Since data aren’t normally distributed, medians were preferred over means as pre- and post-test values. Due to the exploratory analysis and sample size, *p*-values were not corrected for multiple testing.

A *p*-value of <0.05 was considered to be statistically significant. Data was analysed by using the SPSS version 22.0.

### 2.8. Ethical Issues

The project received ethical clearance from the Ethics Committees for Health of the hospitals where the research was conducted, which is in line with the principles of the Helsinki Declaration and later amendments [42]. All permission forms and patient materials were assessed for health literacy and levelled for the target population’s education literacy.

All subjects provided informed consent prior to any evaluation during the enrolment phase. All participants were provided with written and verbal information about the study. Patients were informed that they had the option to withdraw from the study at any time without penalty or censure. All data collecting and management procedures took the participants’ privacy and confidentiality into account. The main researcher (AQ) was not blind for both the intervention and the control group. To ensure equity to access the Hope Intervention Program, the control group participants were offered the opportunity to enrol in the hope activities of the program after the study completion.

## 3. Results

### 3.1. Demographic and Clinical Characteristics

A total of 165 eligible participants were assessed for potential enrolment. Of these, 72 individuals did not meet the inclusion criteria, 29 participants declined to participate mainly due to fatigue, five people had their clinical situation worsen, and three individuals died. A total of 56 patients consented to participate and were enrolled in the study. They were randomly assigned to either the intervention group [IG] (n = 28) or the control group [CG] (n = 28). Since the allocation to the follow-up, several participants dropped out mostly to clinical deterioration and death (see Figure 1).

In a per-protocol analysis, the data are analysed only for patients who reach the study endpoint. Therefore, the characteristics of the participants who completed the protocol originally allocated are summarized in Table 2.

No statistically significant differences were identified between groups in baseline demographic and clinical variables. The equivalence between the two groups was also tested for the hope, comfort, and QoL variables. Groups were similar regarding QoL and comfort, but there were significant differences in hope indexes (*p* = 0.047). As pre-intervention differences were largely due to one individual in CG with lower levels of hope (HHI = 14), this individual was eliminated. Following outlier removal, we obtained the baseline equivalence between both groups in all dependent variables (*p* = 0.074) [see Table 3].

### 3.2. Relationship between Hope, Comfort and QoL in the Sample

The resulting matrix shows positive, moderate and very significant relationships between hope, global comfort and total QoL. The highest correlation was found between hope and QoL (ρ = 0.605, *p* < 0.01) and the lowest between physical QoL and temporality, trust and interconnection (ρ = 0.119, *p* < 0.05). Results also indicate that 22.65% of the variation in comfort is explained by hope, the remaining 77.35% is explained by other factors; and 36.6% of the variation in total QoL is explained by hope, with the remaining 63.4% explained by other factors.

### 3.3. Effect of Intervention

**Figure 2:**
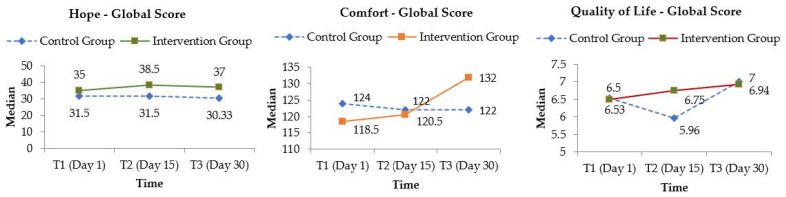
Global median scores evolution of the primary and secondary outcome measures.

The global median scores were graphically represented for each outcome, before and after the intervention in both groups (see Figure 2). Furthermore, Table 4 shows the differences between the intervention and control groups regarding the primary outcome (hope) and the secondary outcomes (comfort and quality of life) at the three data collection points.

#### 3.3.1. Primary Outcome

In the intervention group, there was a significant increase (+3.5; *p* = 0.042) in the total hope scores after the HPP (T1/T2), and this was maintained one month later (T1/T3) (+2.0; *p* = 0.033). The same occurred for positive inner disposition and expectations between T1 and T2. Regarding the HHI subscale scores for temporality, trust and interconnection, the differences were only significant a month after the HPP. Regarding the control group, there were no significant changes in hope scores over time (*p* > 0.05).

#### 3.3.2. Secondary Outcomes

There were no significant differences in comfort scores in either group between T1 and T2. However, there was a significant increase in global comfort median scores in the intervention group one month after the HPP (T1/T3) (+13.5; *p* = 0.018) compared to the CG, which stabilized after T2. However, the increase of relief in the IG extended over time, with significant differences in T2 and T3. One month after HPP, relief was statistically greater than before the program (+0.85; *p* = 0.011) in the participants of the IG, while relief in the CG remained stable.

Regarding the state of ease, there was a statistically significant increase between baseline (T1) and post-intervention (T2) in the CG, but not in the IG. The group submitted to HPP evolved positively and significantly after 15 days (T2). In the CG, the median evolution was decreasing (−0.33) and significant (*p* = 0.045) between T1 and T2 and T1 and T3 (*p* = 0.007).

There was a significant increase in transcendence between T2 (Median = 5.57) and T3 (Median = 5.86; *p* = 0.024) and T1 and T3 (+0.22; *p* = 0.05) in the IG, but a significant decrease in the CG.

Regarding the context of comfort (HCQ), the difference between baseline (T1) and T2 was only significant for the IG in the psychospiritual context (*p* = 0.045), with an immediate increase in average comfort and a difference in the median of +0.44. There were also significant differences between T2 and T3 (*p* = 0.013) in the IG. One month after HPP, the gains in psychospiritual comfort were significant compared to the initial scores (+1.06; *p* = 0.025).

Concerning the evolution of global QoL, no significant differences were identified for the IG between any of the three moments. Despite an increase in the median score in T2 (+0.25) and T3 (+0.19), the average after HPP decreased, corresponding to the lowest value of the three measurements. In the CG, the median evolution was negative between T1 and T2 (−2.01), and then rose significantly in T3 (+3.38).

## 4. Discussion

Comparison between the IG and CG suggests that the HPP had a positive effect on hope over time. The most expressive increases were in the levels of total hope and positive interior disposition and expectations, with significant differences in each period. Regarding temporality, trust and interconnection, differences were only significant one month after the intervention. This can be explained by the high level of hope before the HPP, making it difficult to detect large variations immediately after the intervention program. Even so, the increase was significant when compared to the CG (where levels of hope decreased), attesting to the effect of HPP upon the greater sense of temporality, trust and interconnection over time.

These results support the active and effective role of nurses in promoting the hope of people with advanced chronic illness [23], with an impact in the medium term (one month). Herth [43] had previously confirmed the effect of a hope-promoting program over nine months, with significant effects in all dimensions of hope when compared to a control group. The present study shows that an intervention of shorter duration and involving fewer human and material resources also has positive effects on hope compared to the control group. This result also indicates “a great potential and an added value to the nurses’ role in improving client outcomes through conducting low-intensity psychotherapeutic interventions” [14] (p. 9).

This study was innovative in using a psychosocial supportive hope intervention that differed in terms of dose, composition and the possibility of individualizing the proposed activities. According to Chan et al. [14] “one of the major drawbacks of lengthy programs is their high attrition rate, rendering interventions unfeasible and unsustainable” (p. 3). The previous work by Duggleby et al. [44] was expanded in a home visitation program with three face-to-face sessions led by a nurse, using both audiovisual and written materials to support the activities. Significant differences were obtained in all subscales of HHI, confirming the program’s effectiveness. Indicators were better than those reported by Duggleby et al. [44], who only detected significant effects in total hope and on the temporality and future subscale. A program with the duration of our study can be more effective by offering a wider range of proposed activities accompanied by written support (a practical guide promoting hope).

The tested HPP’s design included a greater number of nurse visits in relation to the “Living with Hope Program” [44]. In terms of effectiveness, human and material resources were committed to ensuring better results. One could question the HPP’s design in this home visitation format, namely whether it would have been more advantageous to use a mix of face-to-face, telephone and e-health interventions, which has proven effects in various areas of health [14,22,45].

The first HPP session, viewing the film “Living with hope”, may have been crucial in shaping the patients’ positive perception of hope. The use of the video has been an integral part of hope-promoting programs [11,22,45], with positive effects on shaping hope. In the published literature, there is evidence of the effectiveness of using video in reducing anxiety related to health situations, but not in improving coping strategies in stressful situations [46]. This study reinforces our knowledge in this area, suggesting positive effects on modelling, but also on self-efficacy. The use of real testimonies seems to have allowed people to identify with the suffering situation and, at the same time, to stimulate their motivation and ability to react positively when facing present problems. In this sense, the video was a resource that promoted hope and empowered patients to deal with situations of suffering through the sharing of information with their peers.

Participants in the IG had a higher global and physical QoL than those of the CG immediately after the HPP. This difference between groups did not remain over time, although there was an increase in both groups. Disease progression and treatments may have prevented better results. However, considering that the instrument can detect changes in 48 h, these results may indicate the need to reinforce hope-promoting interventions and activities in order to obtain gains in QoL over time [47,48].

One of the novelties of this study was the evaluation of the program’s ability to promote hope in comfort. So far, the developed programs have not included the relationship between these variables, although comfort is a major goal of care for people with advanced and progressive chronic illnesses in their end-of-life trajectory [49]. In this context, total comfort involves reviewing life, restoring, and repairing meaningful relationships, hoping for a realistically possible future, and living each day with dignity and peace of mind.

In the HPP, the promotion of hope is associated with interventions and activities directed simultaneously toward comfort in the physical, psycho-spiritual, environmental and socio-cultural contexts. In addition to the proposed hope-focused activities, the program includes a relaxation exercise using guided imagery to reinforce the repertoire of coping strategies, self-control and self-confidence. This constitutes a non-pharmacological tool for symptom control using the ability to transcend the situation [17].

The results indicate that HPP brings long-term gains in terms of overall comfort, relief, ease and transcendence, since the evolution was significant for all these states in the patients in the IG one month after the program. The increase in comfort can be partially explained by the increase in hope, as these variables were positively correlated in the study sample. The increase in comfort was most likely related to the relaxation activity using mental images, since this was performed by all participants. Guided imagination has been identified as a non-pharmacological strategy of symptom control capable of facilitating comfort in a hospital environment [50], although the effects on comfort are not always evident. A recent work by Coelho et al. [50] demonstrated the effectiveness of guided imagery in increasing the comfort of palliative patients. Our results corroborate this increase in comfort over time, adding that guided imagery can increase the comfort of patients at home on a lower dose, assuming at least three sessions.

Notably, there were significant differences between the experimental and control groups in the state of transcendence. This means that, despite the progression of the illness and the effects of their treatments, HPP enabled people to evolve in the discomfort-comfort continuum, increasing their ability to feel more competent and to plan, control their destiny and solve their problems. In the final phase of life, an increase in transcendental comfort can compensate for successive losses of autonomy and control associated with the experience of illness and the real possibility of death in the short term. The fact that patients were mostly Catholic may have influenced transcendence. Believing in life beyond death and giving meaning to suffering (compared with that of Jesus Christ and other models associated with religion) seems to have been decisive for the perception of hope and may also justify the significant gains in comfort in the psycho-spiritual context [17]. This phenomenon can be illustrated by the plastic and written expressions in the practical hope guide of some HPP participants, who identified religious figures as models for their hope.

The active involvement of patients in performing the hope-promoting activities proposed in the guide, which was only possible due to their good functional level, may have facilitated the state of greater harmony, satisfaction and calm necessary for efficient performance, leading to greater transcendental comfort [10]. The evaluation of the patients who carried out the program attests that a predisposition to learn and a positive relational environment facilitated their involvement in the program, providing satisfaction and even leading to the execution of more than one proposed activity without increasing the levels of fatigue.

### 4.1. Study Limitations

Our study had several limitations. Firstly, only one nurse provided the intervention, which might have influenced the results. Secondly, the rapid deterioration in the health status of the participants implied a decrease in the number of patients in each group, which might impact the results. Indeed, participants’ numbers are lower than proposed in the planned power analysis due to recruitment challenges common to end-of-life studies. This reality is unavoidable and is often responsible for the low number of longitudinal studies carried out in this population. The effect of disease symptoms was the main factor behind the study’s dropout rate, with identical rates in both groups. In this study, the HPP was not responsible for patient exhaustion to the point of inducing them to give up due to tiredness. In the per-protocol analysis, data were examined only for those patients who completely adhere to the protocol, as this reduces the statistical power of the study, and the benefits of randomization are lost because the composition of the original groups has been disturbed. Despite the constraints motivated by the loss of subjects, the cases analysed guaranteed group homogeneity, which attests to the reliability of the results. Future studies with mixed methods should address these limitations with larger sample sizes and more diverse settings, including extended and more detailed follow-ups. The HPP should also be extended to include non-terminally ill patients, patients receiving palliative care, and patients with lower levels of functional status [44].

Lastly, and while our clinical trial has not been prospectively registered, this study contains valuable information to improve palliative care and inform future clinical practice. 

### 4.2. Implications

This study can be a useful contribution to updating the International Classification for Nursing Practice (ICNP^®^) catalogue *Palliative Care for Dignified Dying*, namely by incorporating hope-promoting interventions in nursing information systems and in guides for good clinical practice, particularly fostering hope in situations of advanced chronic illness, and by designing training programs for patients, anticipating their needs with regard to hope. In addition, the study might contribute to updating and detailing the hope inspiration interventions listed in the Nursing Interventions Classification [51].

## 5. Conclusions

The HPP may be an effective intervention to increase hope and improve comfort and quality of life among palliative patients. This RCT suggests that is important to create training programs for nurses and nursing students within the scope of hope-promotion in order to promote the development of the personal and professional skills necessary to apply the HPP, both within the scope of palliative care and the context of acute hospitals. The intervention itself should also be developed by exploring other information and communication resources and technologies, namely the telephone, computer and the internet, since the population will tend to be more info-competent in the use of these technologies. The exploitation of these resources can promote empowerment and constitute a useful tool in the positive reinforcement of ill people’s skills and their own hope.

## Figures and Tables

**Table 1:** Investigation protocol.

**Evaluation Time Points**	**T1 (Baseline)**	**RANDOMIZATION**	**Intervention**	**T2 (15 Days after)**	**T3 (after 1 Month)**
Protocol measure and assessment instruments	(1) demographics (gender, age, marital status and social status); (2) clinical characteristics (medical diagnosis, functional status, presence of medical symptoms like pain, fatigue or depression); (3) Herth Hope Index [HHI]; (4) McGill Quality of Life Questionnaire [MQoL]; (5) Hospice Comfort Questionnaire [HCQ].		IG *—Hope Promotion Program (HPP) CG **—Standard palliative care	HHI, MQoL and HCQ	HHI, MQoL and HCQ

* IG—Intervention Group; ** CG—Control Group.

**Table 2:** Participants’ baseline features.

Variables	Intervention Group (n = 12)		Control Group (n = 15)	
**Age** (years) Mean ± (SD)	60.17 (10.83)		60.00 (11.98)	
	**n**	**%**	**n**	**%**
**Sex** (female)	8	29.63	7	25.93
**Married**	10	37.04	12	44.44
**Education** (Primary education)	7	25.93	11	40.74
**Lives with relatives**	11	18.52	13	26.53
**Functional status** (Karnofsky Performance Scale)				
From 60 to 70	0	0	3	11.54
From 80 to 100	12	46.15	11	42.31
**Pain**				
Yes	2	7.41	7	25.93
No	10	37.04	8	29.63
**Fatigue**				
Yes	11	40.74	11	40.74
No	1	3.70	4	14.81
**Depression**				
Yes	5	18.52	6	22.22
No	7	25.93	9	33.33

**Table 3:** Differences in means and results of the Mann-Whitney tests to assess the equivalence between both groups in the variables of hope, comfort and QoL and its dimensions.

	Intervention Group n = 12		Control Group n = 14		U Mann-Whitney (One Tailed *p*)	
	M	SD	M	SD	U	(*p*)
**HHI**	34.166	3.243	31.571	3.715	49.50	0.074
Hope—Fator 1	3.527	0.399	3.226	0.456	50.50	0.082
Hope—Fator 2	3.250	0.399	3.053	0.356	59.00	0.186
**MQoL**	6.817	1.366	6.527	1.583	79.00	0.797
Physical QoL (Quality of Life)	5.667	2.568	5.287	2.536	78.00	0.757
Psychological symptoms	5.459	2.215	5.571	2.588	79.00	0.797
Existential QoL	8.056	1.413	7.595	1.956	71.50	0.520
Support	8.125	1.932	7.714	1.729	69.00	0.436
**HCQ**	118.500	16.473	123.500	15.022	68.00	0.410
Relief	3.541	0.719	3.750	0.667	68.50	0.425
Ease	5.120	0.794	5.261	0.980	71.50	0.518
Transcendence	5.286	0.857	5.520	0.414	75.00	0.641

HHI—Herth Hope Index; MQoL—McGill Quality of Life Questionnaire; HCQ—Hospice Comfort Questionnaire; SD—Standard Deviation.

**Table 4:** Evolution of the total hope, comfort, QoL and its dimensions, in both groups, comparing the three moments of assessment, using Wilcoxon tests.

**Evolution of Hope**		IG * **n = 12**		**CG** **†** **n = 14**	
		**Z**	***p*** **(one tailed *p*)**	**Z**	***p*** **(one tailed *p*)**
Total hope score					
**T1/T2**		−1.536	**0** **.042**	−0.539	0.295
T2/T3		−0.089	0.464	−0.299	0.382
**T1/T3**		−1.841	**0.033**	−0.819	0.206
Factor 1—Temporality, trust and interconnection					
T1/T2		−0.669	0.251	−0.402	0.344
T2/T3		−0.777	0.218	−0.843	0.199
**T1/T3**		−1.730	**0.042**	−0.827	0.204
Factor 2—Positive interior disposition and expectations					
**T1/T2**		−2.146	**0.016**	−0.707	0.240
T2/T3		−1.026	0.152	−0.287	0.387
T1/T3		−1.327	0.092	−0.499	0.309
**Evolution of comfort**		IG * **n = 12**		**CG †** **n = 14**	
		**Z**	***p*** **(one tailed *p*)**	**Z**	***p*** **(one tailed *p*)**
Total comfort score	T1/T2	−0.445	0.328	−1.203	0.114
	**T2/T3**	−2.668	**0.004**	−0.358	0.360
	**T1/T3**	−2.091	**0.018**	−1.203	0.114
Relief	T1/T2	−1.296	0.097	−1.506	0.060
	**T2/T3**	−2.538	**0.005**	−0.059	0.476
	**T1/T3**	−2.268	**0.011**	−0.748	0.227
Ease	**T1/T2**	0.000	0.500	−1.692	**0.045**
	T2/T3	−1.589	0.056	−0.351	0.363
	**T1/T3**	−0.920	0.178	−2.463	**0.007**
Transcendence	**T1/T2**	−0.461	0.322	−2.228	**0.013**
	**T2/T3**	−1.972	**0.024**	−0.632	0.263
	**T1/T3**	−1.643	**0.050**	−2.834	**0.002**
Physical context	T1/T2	−0.713	0.238	−0.223	0.412
	**T2/T3**	−1.793	**0.036**	−0.238	0.406
	T1/T3	−1.382	0.083	−0.870	0.192
Psychospiritual context	**T1/T2**	−1.694	**0.045**	−0.908	0.162
	**T2/T3**	−2.233	**0.013**	−0.920	0.178
	**T1/T3**	−2.807	**0.025**	−0.051	0.479
Environmental context	**T1/T2**	−0.530	0.298	−1.823	**0.034**
	T2/T3	−2.388	0.085	−1.279	0.100
	T1/T3	−1.625	0.052	−0.687	0.246
Sociocultural context	T1/T2	−1.126	0.130	−1.260	0.104
	T2/T3	−0.566	0.285	−0.140	0.444
	T1/T3	−0.313	0.377	−0.938	0.174
**Evolution of QoL (Quality of Life)**		IG * **n = 12**		**CG †** **n = 14**	
		**Z**	***p*** **(one tailed *p*)**	**Z**	***p*** **(one tailed *p*)**
**Total QoL score**	T1/T2	−0.353	0.362	−1.570	0.058
	T2/T3	−0.490	0.312	−1.531	0.063
	T1/T3	−0.000	0.500	−0.903	0.183
Physical QoL	**T1/T2**	−1.493	0.067	−2.169	**0.015**
	**T2/T3**	−2.201	**0.014**	−2.826	**0.002**
	T1/T3	−1.112	0.133	−0.890	0.377
Psychological Symptoms	T1/T2	−1.380	0.084	−0.311	0.377
	T2/T3	−1.068	0.142	−1.290	0.098
	T1/T3	−0.223	0.412	−0.471	0.319
Existential well-being	T1/T2	−0.178	0.429	−0.102	0.459
	**T2/T3**	−0.624	0.266	−2.613	**0.004**
	**T1/T3**	−1.122	0.131	−2.229	**0.013**
Support	T1/T2	−0.422	0.336	−0.823	0.205
	T2/T3	−0.703	0.241	−1.513	0.065
	T1/T3	−0.060	0.475	−0.060	0.475

* T1 and T2 (n = 12); T3 (n = 11). † T1 and T2 (n = 14); T3 (n = 12).

## Data Availability

The data are available upon reasonable request.
